# Macular Oedema in Idiopathic Macular Telangiectasia Type 1 Responsive to Aflibercept but Not Bevacizumab

**DOI:** 10.1155/2014/219792

**Published:** 2014-10-01

**Authors:** O'Sam Shibeeb, Anagha Vaze, Mark Gillies, Timothy Gray

**Affiliations:** ^1^Ophthalmic Research Laboratories, South Australian Institute of Ophthalmology, Hanson Institute Centre for Neurological Diseases, University of Adelaide, Frome Road, Adelaide, SA 5000, Australia; ^2^Department of Ophthalmology and Visual Sciences, Royal Adelaide Hospital, Frome Road, Adelaide, SA 5000, Australia; ^3^Save Sight Institute, The University of Sydney, Sydney, NSW 2006, Australia

## Abstract

We report a patient with macular oedema due to type 1 macular telangiectasia responding to intravitreal aflibercept injection. A 51-year-old man was diagnosed with type 1 idiopathic macular telangiectasia (IMT) in the right eye. The macular oedema was refractory to initial treatment with intravitreal bevacizumab and argon laser photocoagulation. The patient was then treated with intravitreal aflibercept injections, following which the macular oedema was completely resolved and his vision was significantly improved. Intravitreal aflibercept injection appears to improve vision and reduce persistent macular oedema secondary to type 1 IMT and demonstrated promising anatomical and visual outcomes.

## 1. Introduction

IMT refers to a group of retinal vascular anomalies characterised by telangiectatic alterations of the juxtafoveolar capillary network [1]. It was first classified as idiopathic juxtafoveal retinal telangiectasis and subsequently was subdivided into type 1 (aneurysmal telangiectasia) and type 2 (perifoveal telangiectasia-nonproliferative or proliferative) [1, 2]. Type 1 IMT is almost always unilateral and occurs predominantly in middle-aged men. It is characterised by retinal telangiectasia that is commonly confined to the temporal half of the macula in an area of 1-2 disc diameters. Visual loss is mainly caused by cystoid macular oedema (CMO) and formation of hard exudates, which are the hallmark features of the disease [3]. Treatment with laser photocoagulation, photodynamic therapy [4], and intravitreal antivascular endothelial growth factor (anti-VEGF) agents (e.g., bevacizumab) [5–7] has shown mixed results. To the best of our knowledge, this is the first case study describing the use of intravitreal aflibercept for CMO in a case of type 1 IMT which was refractory to bevacizumab and had suboptimal response to macular laser.

## 2. Case Report

**Figure 1:**
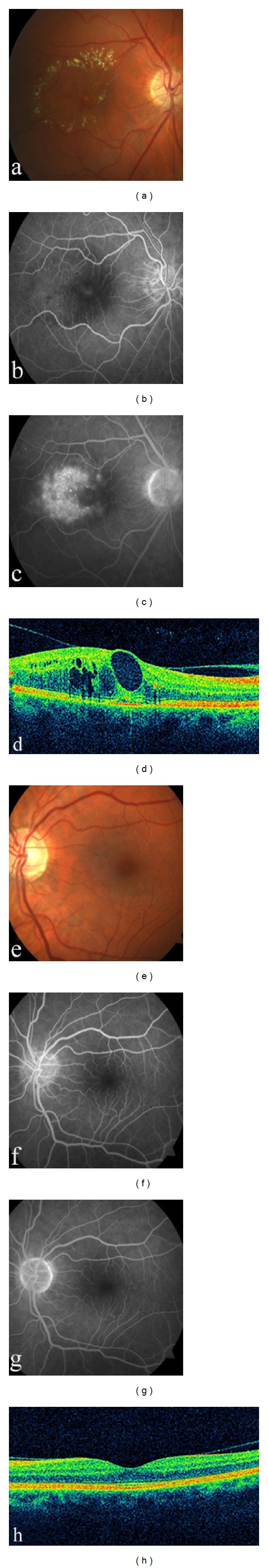
*Before Treatment*. Fundus photograph ((a) right eye, (e) left eye), early phase ((b) right eye, (f) left eye), late phase fluorescein angiography ((c) right eye, (g) left eye), and macular OCT ((d) right eye, (h) left eye).

**Figure 2:**
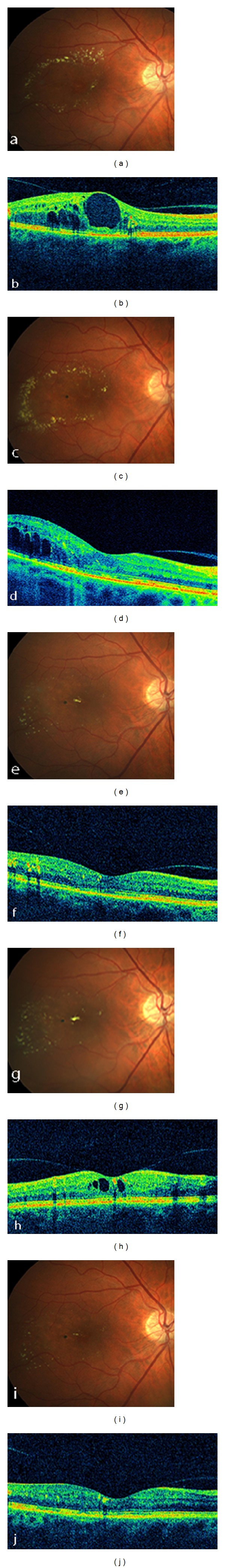
(a)-(b) After 3 monthly* bevacizumab* treatments, (c)-(d) after laser photogaoculation treatment, (e)-(f) after 4 months of* aflibercept* treatment, (g)-(h) after 4 months without treatment, and (i)-(j) after 4 weeks of* aflibercept* injection.

A 51-year-old Caucasian man with no history of diabetes, hypertension, or ischaemic heart disease presented with three to four years of progressive visual loss in the right eye. On examination, his best corrected visual acuity (BCVA) was 6/48 in his right and 6/9.5 in his left eye. Slit lamp biomicroscopy of the anterior segment examination was unremarkable. Fundus examination revealed unilateral parafoveal telangiectasia in the temporal macula of his right eye associated with macular oedema and exudates which was confirmed on optical coherence tomography (OCT). Central macular thickness (CMT) was recorded as 537 *μ*m. Fundus fluorescein angiography (FFA) was performed showing evidence of type 1 IMT in the right eye (Figure 1). Fasting blood glucose and blood pressure were normal. The patient received 3 intravitreal injections of bevacizumab (Avastin) one month apart, but CMT had increased to 594 *μ*m and VA in the right eye declined to 6/60 (Figures 2(a) and 2(b)). Due to the poor response to anti-VEGF treatment, the decision was made to treat the patient with argon laser photocoagulation to the temporal macula. Four months later, there was still substantial macular oedema with underlying microaneurysms and hard exudates and BCVA had further declined to 6/75 (Figures 2(c) and 2(d)). At 4 and 6 months after laser photocoagulation, there was partial improvement in macular oedema with CMT of 375 *μ*m and 315 *μ*m, respectively. However, there were lipid exudates threatening the fovea (Figures 2(e) and 2(f)) and, hence, the patient was offered and consented to treatment using aflibercept. After 4 months of monthly intravitreal injections of aflibercept, there was complete resolution of macular oedema with CMT recorded as 260 *μ*m and recovery of BCVA to 6/24 (Figures 2(e) and 2(f)). Macular oedema recurred with an increase in CMT to 298 *μ*m when left untreated for 4 months (Figures 2(g) and 2(h)), but improved again with CMT recorded as 249 *μ*m 4 weeks following a fifth aflibercept injection (Figures 2(i) and 2(j)) confirming the response to aflibercept.

## 3. Discussion 

Currently, there is no established treatment for type 1 IMT, although retinal photocoagulation has been successful in some cases, reducing the lipid exudates but not always improving vision. It is well known that VEGF stimulates angiogenesis, increases vascular permeability, and is implicated in the formation of abnormal blood vessels in type 2 IMT [8]. Hence, this may provide an explanation as to why type 2 IMT responds to anti-VEGF treatment (e.g., bevacizumab) [9, 10]. However, the exact role of VEGF in type 1 IMT pathogenesis remains unclear and its use in the treatment for this condition has found it to be inconsistent in its outcomes [5, 6]. Aflibercept is a recombinant fusion protein consisting of key human VEGF receptor extracellular domains from receptors 1 and 2 fused to the Fc domain of human IgG1 [11]. Aflibercept binds related growth factors, such as placental growth factors 1 and 2, VEGF-A, and VEGF-B with high affinity [12]. The explanation for the resolution of the CMO in our patient with aflibercept but not bevacizumab remains speculative but may relate to the different binding patterns of these two molecules. Unlike bevacizumab, aflibercept has multiple binding sites, including endothelial cells, pericytes, and the vascular basement membrane [13], potentially accounting for its superior effect in our patient. This case suggests that intravitreal injection of aflibercept can have a role in treating the macular oedema due to type 1 IMT.

In summary, type 1 IMT is a rare retinal condition that can cause significant visual morbidity due to macular oedema. Laser photocoagulation is so far the best documented effective treatment. However, with regard to the risks associated with the laser scars, it would be of great interest to have an effective treatment option without these problems. Aflibercept may be a promising alternate treatment option.
